# eHealth Literacy: Predictors in a Population With Moderate-to-High Cardiovascular Risk

**DOI:** 10.2196/humanfactors.6217

**Published:** 2017-01-27

**Authors:** Sarah S Richtering, Karice Hyun, Lis Neubeck, Genevieve Coorey, John Chalmers, Tim Usherwood, David Peiris, Clara K Chow, Julie Redfern

**Affiliations:** ^1^ The George Institute for Global Health Sydney Australia; ^2^ Hôpitaux Universitaires de Genève Université de Genève Geneva Switzerland; ^3^ Sydney Medical School University of Sydney Sydney Australia; ^4^ School of Health and Social Care Edinburgh Napier University Edinburgh United Kingdom; ^5^ Sydney Nursing School Charles Perkin Centre University of Sydney Sydney Australia; ^6^ Westmead Hospital Sydney Australia

**Keywords:** eHealth, socioeconomic factors, health literacy, cardiovascular system, chronic disease, Internet

## Abstract

**Background:**

Electronic health (eHealth) literacy is a growing area of research parallel to the ongoing development of eHealth interventions. There is, however, little and conflicting information regarding the factors that influence eHealth literacy, notably in chronic disease. We are similarly ill-informed about the relationship between eHealth and health literacy, 2 related yet distinct health-related literacies.

**Objective:**

The aim of our study was to investigate the demographic, socioeconomic, technology use, and health literacy predictors of eHealth literacy in a population with moderate-to-high cardiovascular risk.

**Methods:**

Demographic and socioeconomic data were collected from 453 participants of the CONNECT (Consumer Navigation of Electronic Cardiovascular Tools) study, which included age, gender, education, income, cardiovascular-related polypharmacy, private health care, main electronic device use, and time spent on the Internet. Participants also completed an eHealth Literacy Scale (eHEALS) and a Health Literacy Questionnaire (HLQ). Univariate analyses were performed to compare patient demographic and socioeconomic characteristics between the low (eHEALS<26) and high (eHEALS≥26) eHealth literacy groups. To then determine the predictors of low eHealth literacy, multiple-adjusted generalized estimating equation logistic regression model was used. This technique was also used to examine the correlation between eHealth literacy and health literacy for 4 predefined literacy themes: navigating resources, skills to use resources, usefulness for oneself, and critical evaluation.

**Results:**

The univariate analysis showed that patients with lower eHealth literacy were older (68 years vs 66 years, *P=*.01), had lower level of education (*P=*.007), and spent less time on the Internet (*P*<.001). However, multiple-adjusted generalized estimating equation logistic regression model demonstrated that only the time spent on the Internet (*P=*.01) was associated with the level of eHealth literacy. Regarding the comparison between the eHEALS items and HLQ scales, a positive linear relationship was found for the themes “usefulness for oneself” (*P*=.049) and “critical evaluation” (*P*=.01).

**Conclusions:**

This study shows the importance of evaluating patients’ familiarity with the Internet as reflected, in part, by the time spent on the Internet. It also shows the importance of specifically assessing eHealth literacy in conjunction with a health literacy assessment in order to assess patients’ navigational knowledge and skills using the Internet, specific to the use of eHealth applications.

## Introduction

People are increasingly managing their health with the aid of electronic tools [1,2]. This requires an understanding of their condition as well as the skills to effectively use and navigate the devices available [1,3]. This skill is referred to as electronic Health (eHealth) literacy and is a growing area of research parallel to the increasing development and use of eHealth interventions. Although access to the Internet is fairly widespread [4], eHealth resources are constantly evolving and require an ongoing adaptation by their users [5,6]. To ensure that a patient is able to use the available resources effectively, it therefore becomes necessary to assess their eHealth literacy and identify its determining factors in order to improve access and usability.

Despite its increasing importance, to date there has been limited investigation into the demographic, socioeconomic, and technology use determinants of eHealth literacy. In healthy adults, lower age and higher education correlate to higher eHealth Literacy [1,7-9], as does higher Internet use and number of electronic devices used [2]. Likewise in younger adults, eHealth literacy correlates positively to education, electronic device use, and Internet use [9] with increasing age and duration of illness having a negative impact on eHealth literacy. In underserved populations, active Internet use and urban dwelling are associated with increased eHealth literacy, as is higher income [8], which is not the case in a general population study [9]. Conversely, a study examining the success of an eHealth intervention in elderly patients undergoing cardiac rehabilitation found that age and gender had no influence on eHealth literacy [10]. These diverging results demonstrate the difficulty in identifying generalizable predictors of eHealth literacy to all populations.

People with cardiovascular disease (CVD) are required to self-manage many aspects of their condition, and this requires a minimal level of health literacy. Health literacy is defined as the “knowledge, motivation, and competences to access, understand, appraise, and apply health information in order to make judgments and take decisions in everyday life concerning health care” [11]. In a population with CVD, poor health literacy was found to be associated with decreased health status [12]. Medication adherence is a strong determinant of health outcomes in patients with CVD and has been shown to improve through active patient education and electronically based reminders [13]. Likewise, eHealth interventions have shown promising results toward increasing health literacy [8,14]. How health literacy and eHealth literacy are correlated in a population with cardiovascular risk has not been examined. This was the first study to examine this relationship as well as the demographic, socioeconomic, technology use, and health literacy predictors of eHealth literacy in a population with moderate-to-high cardiovascular risk.

## Methods

### Design

People diagnosed with or at risk for CVD were assessed to explore the relationship between demographic characteristics, socioeconomic factors, use of technology, health literacy, and eHealth literacy. The sample consisted of 453 participants in the Consumer Navigation of Electronic Cardiovascular Tools (CONNECT) Study [15]. All participants provided written, informed consent, and ethical approval was obtained from the Human Research Ethics Committee (Project number 2013/716).

### Recruitment

The CONNECT study methods and participant recruitment processes are detailed elsewhere [15]. In brief, CONNECT is an ongoing randomized controlled trial examining whether an eHealth strategy improves risk factor control when compared with usual health care in patients with or at risk for CVD. Participants were recruited via Australian primary care practices. The inclusion criteria were as follows: aged 18 years or older, have access to the Internet at least once a month, and have moderate-to-high risk for a CVD event. Moderate-to-high CVD risk was defined as (1) ≥10% 5-year CVD risk using Framingham risk equation; (2) a clinically high-risk condition (Aboriginal or Torres Strait Islander >75 years, diabetes and >60 years, diabetes and albuminuria, estimated glomerular filtration rate <45 mL/min, systolic blood pressure ≥180 mmHg, diastolic blood pressure ≥110 mmHg, total cholesterol >7.5 mmol); and (c) an established CVD diagnosis (ischemic heart disease, stroke or transient ischemic attack, peripheral vascular disease). Participants with an insufficient level of English proficiency or severe intellectual disabilities were excluded. At baseline, demographic and socioeconomic data were collected, and participants completed eHealth and health literacy questionnaires (HLQs).

### Assessment of eHealth Literacy: eHealth Literacy Scale

The eHealth Literacy Scale (eHEALS) is one of the very few existing scales assessing eHealth literacy. It comprises 8 items scored on a 5-point Likert scale and aims to reflect the individuals’ own perception of their knowledge and skills at using eHealth information [16,17]. The final result is the sum of all items ranging from 8 to 40 with higher scores reflecting a higher level of eHealth literacy. The validity and reliability of eHEALS has been demonstrated in various health conditions [14,18] and ages [5,19,20] and has been translated into many languages [21-23]. As recommended by the developers, 2 questions were added prior to the 8 items to capture the participants’ opinion about the importance and usefulness of eHealth [7,14]. Following other studies with similar target populations, the cutoff for high eHealth literacy was set at 26 [2,7,9,14,21,23]. High eHealth literacy level (eHEALS≥26 out of 40) and low eHealth literacy levels (eHEALS<26) were thus compared for predefined demographic and socioeconomic factors.

### Assessment of Health Literacy: HLQ

The HLQ was used to assess health literacy. It comprises 9 independent scales that assess distinct aspects of health literacy and aims to measure an individual’s capacity at effectively using health information and services [24]. Each scale is composed of 4 to 6 items and is scored on a 4- or 5-point Likert scale [24]. The score for each scale is the mean score of its items where higher scores indicate higher health literacy levels [24,25] with no fixed values distinguishing high or low levels. It was developed to provide a comprehensive assessment of health literacy compared with other existing tools [26], has demonstrated good construct validity, and has been widely translated [27-29].

### Comparison Between HLQ and eHEALS

In order to examine the relationship between health and eHealth literacy, we undertook a process of matching HLQ scales to eHEALS items by grouping related items with similar themes (Table 1). eHEALS items 1, 2, and 3 related to Internet navigational skills and were thus grouped together. Likewise, items 6 and 7 both related to evaluation of resources found on the Internet. Items 4 and 5 represented distinct aspects of eHealth literacy and were therefore not grouped. Only item 8 of the eHEALS (“I feel confident in using information from the Internet to make health decisions”) was excluded as there was no HLQ scale that comparably assessed the confidence related to using health resources. Four key aspects of eHealth and health literacy were thus defined, and mean scores for items or item groups were then derived for each patient. For “navigating resources” and “skills to use resources,” the HLQ scales ranged from 1 to 5, and for “usefulness for oneself” and “critical evaluation,” they ranged from 1 to 4. This process was performed iteratively and via consensus between experts in clinical practice, research, and statistical analysis (Table 1).

**Table 1:** Matching eHEALS (eHealth Literacy Scale) [16] items to the HLQ (Health literacy Questionnaire) [24].

Areas	eHEALS^a^ questions	HLQ^b^ subscales
Navigating resources	Item 1: I know what health resources are available on the Internet Item 2: I know where to find helpful health resources on the Internet Item 3: I know how to find helpful resources on the Internet	Navigating the health care system (range 1-5)
Skills to use resources	Item 4: I know how to use Internet to answer my questions about health	Ability to find good health information (range 1-5)
Usefulness for oneself	Item 5: I know how to use the health information I find on the Internet to help me	Having sufficient information to manage my health (range 1-4)
Critical evaluation	Item 6: I have the skills I need to evaluate the health resources I find on the Internet Item 7: I can tell high-quality health resources from low quality health resources on the Internet	Appraisal of health information (range 1-4)

^a^eHEALS: eHealth Literacy Scale.

^b^HLQ: Health Literacy Questionnaire.

### Statistical Analysis

Univariate analyses were performed to compare patient demographic, socioeconomic, and technology use characteristics between the low (eHEALS<26) and high (eHEALS≥26) eHealth literacy groups. Chi-square test was used to compare the categorical variables, and independent *t* test was used to compare the means between the 2 groups. To determine predictors of low eHealth literacy, multiple-adjusted generalized estimating equation logistic regression model was used. Independent predictors included in the model were gender (female or male), age (<65 or 65-70 or >70 years), education (≤secondary or university or technical or vocational training), income (<Aus $1000 or Aus $1000-2000 or > Aus $2000 per week), CVD-related polypharmacy (active consumption of >3 medications related to CVD), private health care (yes or no), main electronic device used (desktop or laptop or mobile phone or tablet), and time spent on the Internet on any device (≤1 hour or >1 hour per day). These variables were included regardless of the statistical significance in the univariable comparison due to their clinical significance in relation to eHealth literacy. This analysis adjusted for the clustering effect of primary health care practices. The derived odds ratios (ORs) and corresponding 95% CIs were plotted in a forest plot. An adjusted analysis using the eHEALS score as a continuous variable was also done to see whether other predictors emerged. To test for the correlation between eHealth literacy and health literacy for the 4 literacy themes, multiple-adjusted generalized estimating equation linear regression models were used for each of the themes. The dependent variable, eHEALS score, was in a continuous form, and the corresponding continuous HLQ score was included in the model with the aforementioned covariates. Data were analyzed using SAS version 9.4 for Windows (SAS Institute Inc).

## Results

### Principal Findings

In total, 453 participants were included in the analysis; 1 was excluded due to an incomplete eHEALS (Table 2). The mean age of the sample was 67 years (range: 45-89; standard deviation, SD 8.0), 75.9% (344/453) were male, 89.0% (403/453) were white, and 80.4% (364/453) were either married or in a de facto relationship. The sample was overall well educated (53.4%, 242/453; had undergraduate or postgraduate degree), and 81.0% (367/453) had private health insurance. Over half the sample stated that the Internet was useful or very useful to make decisions regarding health (n=257), and that it was either important or very important for them to be able to access health resources on the Internet (n=267). The mean eHEALS score was 27.2 (range: 8-40; SD 6.59), which was in the high eHealth literacy range (≥26). A total of 175 participants had an eHEALS score within half an SD value of 26. The HLQ scores were 4.12 (SD 0.53) and 4.07 (SD 0.54) out of 5 for “navigating the health care system” and “ability to find good health information,” respectively and 2.92 (SD 0.46) and 2.79 (SD 0.51) out of 4 for “having sufficient information to manage my health” and “appraisal of health information,” respectively. When we compared the cohort with low (n=154) and high (n=299) eHealth literacy, those with high eHealth literacy were more likely to be younger, have a higher level of education, and spend more time on the Internet (Table 2). The results were similar when using a continuous variable.

**Table 2:** Univariable comparison of demographic, socioeconomic, and technology use factors in eHealth literacy (analysis adjusted for the clustering effect of primary health care practices).

Variable		Low eHealth literacy (eHEALS^a^<26) (n=154), n (%)	High eHealth literacy (eHEALS≥26) (n=299), n (%)	Overall (N=453) n (%)	*P* value
Male		124 (80.5)	220 (73. 6)	344 (75.9)	.10
**Age in years, mean (SD)**		68 (8)	66 (8)	67 (8)	.01
	Age <65 years, n (%)	49 (32)	121 (40.5)	170 (37.5)	.02
	Age 65-70 years, n (%) 50 (32)	108 (36.1)	158 (34.9)		
	Age >70 years, n (%)	55 (36)	70 (23)	125 (27.6)	
History of coronary heart disease, n (%)		49 (32)	92 (31)	141 (31.1)	.82
Taking >3 CVD^b^ medications		30 (19)	73 (24)	103 (22.7)	.24
**Education level, n (%)**					
	None, primary, or secondary	54 (35)	67 (22)	121 (26.7)	.007
	Technical or vocational training	32 (21)	58 (19)	90 (20)	
	Undergraduate or postgraduate	68 (44)	174 (58.2)	242 (53.4)	
**Income (in Aus $ per week), n (%)**					
	<1000	30 (19)	56 (19)	86 (20)	.55
	1000-2000	54 (35)	126 (42.1)	180 (39.7)	
	>2000	45 (29)	82 (27)	127 (28.0)	
Private insurance, n (%)		118 (76.6)	249 (83.3)	367 (81.0)	.09
**Main device used to access the Internet, n (%)**					
	Desktop computer	62 (40)	132 (44.1)	194 (42.8)	.53
	Laptop	59 (38)	100 (33.4)	159 (35.1)	
	Mobile phone or tablet	31 (20)	67 (22)	98 (22)	
Spends >1 hour on Internet per day		56 (36)	179 (60.0)	235 (55.8)	<.001
eHEALS score, mean (SD)		19.89 (4.909)	30.96	27.2	<.001

^a^eHEALS: eHealth Literacy Scale.

^b^CVD: cardiovascular disease.

### Predictors of Low eHealth Literacy

**Figure 1:**
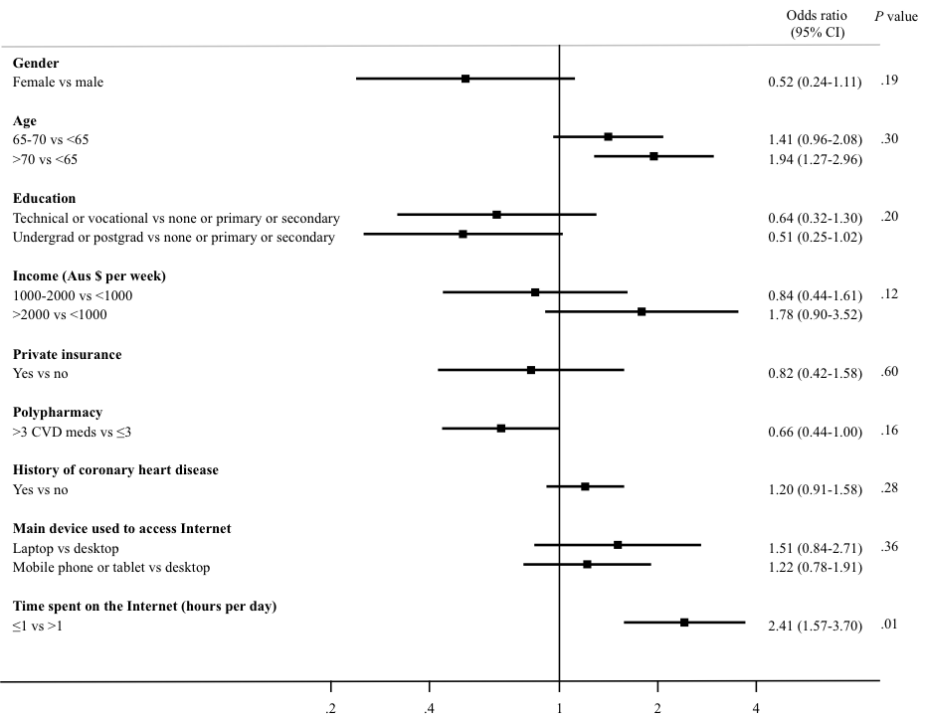
Predictors of low eHealth literacy (defined as eHEALS score <26 out of 40; analysis adjusted for the demographic, socioeconomic, and technology use predictors). CVD: Cardiovascular disease; eHEALS: eHealth Literacy Scale.

The univariate analysis showed that patients with lower eHealth literacy were older (68 years vs 66 years, *P=*.02), had lower level of education (*P=*.007), and spent less time on the Internet (*P*<.001; Table 2). Gender, CVD-related polypharmacy, history of coronary heart disease, income categories, and main device used to access the Internet numbers were similar between the 2 groups. After adjustment for demographic, socioeconomic, and technology use, only the time spent on the Internet (*P=*.01) was associated with the level of eHealth literacy (Figure 1). Participants who spent less than or equal to 1 hour on the Internet per day were 2.45 times more likely to have low eHealth literacy compared with those who spent more than 1 hour per day. Conversely, age (*P=*.26), gender (*P=*.18), education (*P=*.19), income (*P=*.15), CVD-related polypharmacy (*P=*.22), private insurance (*P=*.47), and main device used to access the Internet (*P=*.30) did not achieve statistical significance.

### eHEALS Versus HLQ

After adjustment for demographic, socioeconomic factors, and technology use, a positive linear relationship was found between the eHEALS items and HLQ scales for the themes “usefulness for oneself” (*P*=.049) and “critical evaluation” (*P*=.01; Table 3). For every point gained in the HLQ scale “Having sufficient information to manage my health” (range 1-4), there was a gain of 0.5 in eHEALS item 5 (“I know how to use the health information I find on the Internet to help me”). Similarly, an increment of every point in the HLQ scale “appraisal of health information” (range 1-5) corresponded to an increment of 0.80 increase in items 6 and 7. However, for “navigating resources” (*P*=.08) and “skills to use resources” (*P*=.06), the 2 scales were not well correlated.

**Table 3:** Comparison between eHealth Literacy Scale (eHEALS) [16] and the Health Literacy Questionnaire (HLQ) [24].

Themes^a^	Beta coefficient of HLQ^b^ score (SE^c^)	*P* value
Navigating resources	0.3117 (0.0970)	.08
Skills to use resources	0.4108 (0.0977)	.06
Usefulness for oneself	0.5222 (0.1439)	.049
Critical evaluation	0.7955 (0.0844)	.01

^a^The analysis was adjusted for the demographic, socioeconomic, and technology use predictors.

^b^HLQ: Health Literacy Questionnaire.

^c^SE: Standard error.

## Discussion

### Principal Findings

In this sample of participants with moderate-to-high CVD risk, over half felt that the Internet was useful for health, and that access to health information via the Internet was important to them. With regards to the demographic and socioeconomic predictors, age, gender, education, income, CVD-related polypharmacy, private insurance, and main device used to access the Internet were not statistically significant. Only the total time spent on the Internet per day, the only modifiable predictor that was tested, significantly determined eHealth literacy level, independently of the device used. These results implied that the level of eHealth literacy was directly correlated to the time spent on the Internet and was independent of nonmodifiable personal or socioeconomic characteristics. With regards to the relationship between eHealth and health literacy, only participants’ perceptions of the usefulness of electronic resources for themselves and their critical evaluation were associated; navigation skills and confidence were not.

### Comparison With Prior Work

Prior studies using the eHEALS have shown conflicting results regarding the demographic and socioeconomic determinants of eHealth literacy. Increasing age, for example, was found to predict lower eHEALS scores in healthy older adults [2] and underserved populations [8], whereas it was not found to be predictive in people with lung cancer [14] and rheumatic disease [23]. Similarly, education level was shown to increase eHealth literacy in older populations [2,8] and in people with lung cancer [14]. However this finding was not shown in other studies examining populations with chronic diseases [23,30]. Findings on the socioeconomic determinants of eHealth literacy are also contradictory with gender, marital status, and income being unrelated to eHEALS scores in older populations [2] but influential in a population with colorectal cancer [30]. The sole predictor in most prior studies using the eHEALS, [7,8,14,19,23], as in this study, was frequency of the Internet use. As for the relationship between eHealth and health literacy, a systematic review found that lower health literacy was predictive of lower eHealth literacy levels [8].

Given the diverging findings in prior studies with chronic diseases, the results of this study supported that the demographic and socioeconomic predictors of eHealth literacy were largely population dependent. Furthermore, this study provided further evidence that increased Internet use predicts higher eHealth literacy. The 2 aspects common to both the eHEALS and HLQ were “usefulness for oneself” and “critical evaluation,” which both related to a patient’s personal interpretation of the health information they were confronted to. This interpretation is independent of the knowledge and skills needed to effectively use electronic resources, which are very specific to eHealth and not necessarily addressed in a health literacy scale. The findings of this study reinforced the importance of evaluating patients’ knowledge and access to electronic information through an eHealth literacy assessment alongside a health literacy assessment. By assessing these 2 types of literacy before implementing an eHealth intervention, participants who had a low level of eHealth literacy could thereby benefit from education in using electronic resources.

### Strengths

This was the first study to examine demographic, socioeconomic, and technology-related determinants of eHealth literacy in a population with moderate-to-high CVD risk. By identifying patient characteristics that influence access to eHealth resources, health management and patient empowerment could be improved when using electronic resources. Although most predictors such as age, gender, education, and income are not modifiable, this study showed that the prevailing predictor of eHealth literacy was total time spent on the Internet, consistent with prior eHealth literacy research. Other studies do, however, underline that it is specifically time spent using Web-based health-related resources that increases eHealth literacy and not the time spent on the Internet in general [8]. Assessing time spent on the Internet is a simple and efficient way of determining the potential appropriateness of an eHealth intervention for a given patient. This was also the first study correlating an eHealth literacy scale with a health literacy scale. This comparison demonstrated the differences in these 2 related yet distinct types of literacies and the importance in assessing them individually and simultaneously. Although a patient may have access to the Internet, they require the skills to use it in an effective and beneficial way [7,14]. Further research with different scales and study populations is required, but this study nonetheless highlighted the importance of evaluating eHealth literacy aspects, which are not necessarily covered by a health literacy assessment. Future research is also needed to explore the health (including quality of life) outcomes associated with varying levels of eHealth literacy and the amount of time patients spend using the Internet. In addition, future research could clarify the value and importance of assessing eHealth literacy on a validated scale compared with asking the question of time spent on the Internet at baseline. Although we chose to use specific assessment tools for health literacy, this is a growing area and future research can also make comparisons with alternative tools.

### Limitations

This study had several limitations. First, the study population was largely male (75.9%, 344/453), white (89.0%, 403/453), and well educated (53.6%, 243/453; had a graduate education), and all had access to the Internet. Furthermore, people who agreed to participate were likely to be more motivated and interested in their health management, which might have introduced an element of selection bias. Although this limited the generalizability of the findings to all populations with and at risk for CVD, an ongoing Australian CVD registry found 70% of male prevalence [31] and that 86% of Australians had access to the Internet in 2014-2015 [32]. Second, this study did not ask participants the purpose of their using the Internet in the time they spent on it. The increasing use of Web-based resources for professional reasons could constitute a considerable portion of the time spent on the Internet, even in this older population. This is particularly relevant because, as previously mentioned, it is the frequency of use of health-related information that increases eHealth literacy. Furthermore, this study did not assess patient’s ability to determine the quality of Web-based resources, and a recent study looking at the quality of health information related to weight loss found that the content of more readily available information on search engines was suboptimal [33]. Another limitation was that the eHEALS reflects self-perception of eHealth literacy and not actual skill. This is particularly relevant, as the only study that examined the relationship between the eHEALS score and health-related Internet skills in a sample of people with rheumatic disease found that they were not correlated [23]. It remains, however, the only study to demonstrate this finding, and further research is required to investigate it more fully. Finally, to truly assess the importance of an eHEALS score, intervention studies to increase eHealth literacy should be conducted to assess their impact on clinically important outcomes.

### Conclusions

As Internet-based eHealth interventions are increasingly being developed to facilitate patients’ health management, it becomes essential to gain an understanding of their eHealth literacy and identify its predictors. If users do not have an adequate level of eHealth literacy, certain Internet-based eHealth interventions could be compromised. This study has shown the importance of evaluating patients’ familiarity with the Internet, as reflected, in part, by the time spent on the Internet, to improve their eHealth literacy. It has also shown the importance of specifically assessing eHealth literacy in conjunction with a health literacy assessment in order to assess patients’ navigational knowledge and skills using the Internet. Although related, eHealth literacy requires knowledge of electronic resources and abilities to use them, which are distinct from purely an understanding of health or health literacy.

## Abbreviations

CONNECT: Consumer Navigation of Electronic Cardiovascular Tools
CVD: cardiovascular disease
eHealth: electronic Health
eHEALS: eHealth Literacy Scale
HLQ: Health Literacy Questionnaire
